# Supplementary material for: A set of multi-entry identification keys to African frugivorous flies (Diptera, Tephritidae)
Source: Zookeys. 2014 Jul 24;(428):97–108. doi: 10.3897/zookeys.428.7366 (PMC4143993; doi:10.3897/zookeys.428.7366)
Supplement: Supplementary material 6 — Key to Ceratitis [file zookeys-428-097-s006.zip › SF6_ZooKeys_key to Ceratitis/key/SF6_ZooKeys_key to Ceratitis/Media/Html/Ceratitis colae .htm]

Ceratitis (Pterandrus) colae Silvestri


***Ceratitis*** **(*Pterandrus*) *colae*
Silvestri**

*Ceratitis* *colae* Silvestri, 1913: 63.

Body
length: 4.95 (4.30-5.50) mm; wing length: 5.19 (4.25-5.90) mm.

Male

Head: Antenna yellowish orange to orange. First flagellomere
with dorsal margin sometimes brown; 2-3 times as long as pedicel. Arista with
moderately long rays; ventral rays shorter and sparser than dorsal rays,
especially basally. Frons convex or flat; yellow to orange; with short
scattered setulae distinctly darker than frons. Frontal setae well developed. Face
yellow; gena brown, partly continued along ventral margin of face but not in
median part; mouthparts with apex partly darkened but not completely black (as
in lepida). Genal seta and setulae dark, well developed.

Thorax:
Postpronotal lobe white, without spot. Mesonotum: ground color dark gray with
silvery shine; with streaks and darker markings but without distinct spots or
clearly defined stripes, except prescutellar yellow markings, which are
separate. Scapular setae dark. Scutellum white, basally without spots, apically
with three separate black spots, extending to half-way. Anepisternum on ventral
half brownish black; setulae on ventral half black, ventral to
anepisternal seta with longer black setulae.

Legs:
Yellow except where otherwise noted; setation typical for subgenus, mainly
dark. Foreleg: coxa brown, occasionally almost black; femur anteriorly with
contrasting black/white pattern, basally pale yellow; white spot turns silverly
when viewed from certain angle, anteroventrally with bush of short dark setulae
in median half; posteriorly slightly darkened along dorsal and ventral margin;
with dispersed and poorly developed bush of long dark setulae along entire
length, posterodorsal setulae longer; ventral setae dark; tibia anteriorly
slightly darkened. Midleg: femur anteriorly yellow to yellowish brown;
anteroventrally with conspicuous silvery patch, distally with inconspicuous
one; ventrally with dark feathering along entire length except for interruption
(6-7 setae wide) in median part; tibia broadened; yellow, silvery shine when
viewed from certain angle; with black feathering dorsally along distal 0.8 and
ventrally along distal 0.66, anteriorly with dispersed short setulae, in front
of dorsal row forming an irregular second one. Hindleg: femur at apical 0.25
with longer setulae dorsally and ventrally.

Wing:
Bands brown or yellowish brown. Interruption between marginal and discal bands
near vein R_1_ clear and complete; cubital band free; medial band
absent; crossvein R-M proximal to middle of discal cell. Apex of vein R_1_
distal to level of crossvein R-M. Crossvein DM-Cu oblique anterobasally, or
straight.

Abdomen:
Mostly yellow. Tergite 1 with small brown patches at posterior margin. Tergites
2 and 4 with silvery transverse band along posterior half. Tergite 3 with brown
transverse band along� posterior 0.33, sometimes interrupted in middle.
Tergite 5 with yellowish brown patches along anterior part and weak silvery
band posteriorly. Sometimes abdomen with general darker brown appearance.
Setation and banding typical for subgenus. Male epandrium in lateral view with
posterior lobe of lateral surstylus elongate, apical end curved.

Female

As
male except for the following characters: Legs without feathering; yellow,
femora darker; forefemur posteroventrally with dark pilosity. Wing usually with
darker banding. Abdominal tergite 5 with distinct silvery band posteriorly.
Oviscape almost as long as preabdomen. Aculeus at least eight times longer than
wide; tip with distinct apical indentation and lateral margin slightly sinuous.

(Description
after De Meyer & Freidberg, 2006)
